# Editorial

**Published:** 1892-06

**Authors:** 


### HOMŒOPATHIC PHYSICIAN,

A MONTHLY JOURNAL OF

HOMŒOPATHIC MATERIA MEDICA AND CLINICAL MEDICINE.

"If our school ever gives up the strict inductive method of Hahnemann, we are lost, and deserve only to be mentioned as a caricature in the history of medicine."—CONSTANTINE HERING.

Vol. XII. JUNE, 1892. No. 6.

### EDITORIAL.

SHOULD THE PATIENT BE TOLD WHAT REMEDY HE IS TAKING?—This is a question frequently asked and there is some diversity of opinion about the answer to it. Many physicians from desire to please the patient and so retain his good-will, tell what remedy is administered. The patients are with few exceptions decidedly of opinion that they should be informed what remedy they are taking. They are thus enabled to set up their judgment against that of the physician. This they seldom fail to do, thereby interfering with the course of treatment, baffling the physician, and preventing his exercising his judgment with that freedom which is so necessary to successful prescribing.

There are two principal reasons why patients should be kept in ignorance of the treatment.

The first of these reasons may be found in the results of the action of the similar remedy upon the individual.

When a remedy is truly the simillimum to any given sick condition, it not only cures the case but it so effectually changes its character that it may be said to fortify the system against it and thus render it inert if prescribed again in a short time after it has once produced its effect. In this respect it resembles the action of vaccine virus.

All homœopathists who prescribe the similar remedy must have observed this phenomenon.

Patients do not understand it, and if they have learned that a certain cure was produced by a certain remedy, they will procure a large supply of the medicine and the next time they are sick in any way even remotely resembling the previous illness, they begin at once to dose themselves with it, complicating the case without of course producing any relief, and so when at last they are compelled to summon the doctor, the latter finds much more difficulty in selecting the simillimum than he would otherwise, if the case had not been tampered with by the patient.

The second reason for not informing the patient the name of the medicine lies in the fact that, as stated at the beginning of this article, the patients set up their own judgment in opposition to that of the physician, and do not hesitate to take any remedy that suggests itself to them whilst still under treatment.

How often will women who have learned the virtues of Pulsatilla, in ailments peculiar to their sex take unauthorized doses of that medicament whilst still under the care of a physician for that very complaint! The writer has wasted many hours, earnestly seeking the simillimum in the case of a certain woman and having found it he has administered it, and then in two or three days has been astounded to find his efforts thwarted, and his labor wasted by the interference of that woman who has taken Pulsatilla because she "thought she needed it." Every physician has no doubt had this experience.

The writer had a case of a man who had a peculiar form of nervousness for which Ignatia was given with most brilliant effect. The patient inquired the name of the remedy and the doctor thoughtlessly gave it. The patient at once bought a good supply of the medicine with which he dosed himself whether he was under treatment or not, without consulting the doctor. If he wrote a note for medicine he did not fail to add a request that Ignatia be put into the prescription "for nervousness."

The family medicine-chest very materially increases this difficulty of the physician in managing his cases of illness successfully.

The late Dr. Lippe never would tell the name of a remedy he was prescribing if he could possibly avoid it. He would give strict injunctions to the writer of this article in the numerous cases which the latter attended as the assistant of Dr. Lippe, on no account to give the patient any information concerning the remedy. "You are not supposed to know anything about it, *what* remedy I am giving," he would say when speaking on this point. He was also a determined enemy of the family medicine-chest; and he was frequently heard indulging his denunciations of that potent source of interference with the treatment of a case.

The old school of medicine has been seriously embarrassed by this same difficulty of the patients' learning the character of the prescriptions, and setting up their own judgment in regard to them. Herein lies the origin of the practice of druggists "renewing" prescriptions which they have originally compounded.

This custom has been a great source of complaint of physicians against the druggists, and a few years since gave rise to a ludicrous controversy in this city between the two parties.

The doctors had been supplying themselves with pocket medicine-cases gotten up for them by certain well-known pharmacists in imitation of the homœopathists, and the druggists, fearing the effect of these on their trade, tried to induce the physicians to abandon the use of them by promising not to make up any more "renewals" of prescriptions. Their efforts, however, failed.

Some years ago the peasants of a certain part of France become so much incensed against the prescribing of Mercury by physicians that they would refuse to take any prescription which they suspected to contain it. To circumvent them the doctors were obliged to resort to the subterfuge of calling it "Metallum" when writing a prescription.

In the old school the interference of the patient in prescribing for himself without the consent of the physician means generally not only the loss of a fee, but the poisoning of the patient as well. In the new school it means the complicating of the case, and the baffling of the physician in his efforts to find the simillimum, and so prolonging the case. The most important step in treating a case is the *first* step; the selecting the first remedy. If that be the true simillimum all the rest is plain sailing. If, however, the patient make the first prescription, the result is complications and difficulties without number, and the prolongation of the case. Therefore, it is not well to inform the patient the name of his remedy.

---

WELLS ON INTERMITTENT FEVER.—Our subscribers will have noticed that no more fascicles of this book have been published. The reason is that, except a page or two yet to come, it is finished. But it has no index. This is now being prepared, and then we shall consider the work complete. The *Repertory* of Bœnninghausen it is not necessary to publish over again, as the profession are already supplied with the excellent translation of Dr. Augustus Korndœrfer.

